# Changes in Maternal Serum Transforming Growth Factor Beta-1 during Pregnancy: A Cross-Sectional Study

**DOI:** 10.1155/2013/318464

**Published:** 2013-11-18

**Authors:** Mandeep Singh, Ngozi C. Orazulike, Jill Ashmore, Justin C. Konje

**Affiliations:** ^1^Women's Hospital, Kensington Building, Leicester Royal Infirmary, Leicester LE1 5WW, UK; ^2^University of Port Harcourt Teaching Hospital, Rivers State, P.M.B 6713, Nigeria; ^3^Reproductive Sciences Section, Department of Cancer Studies and Molecular Medicine, Robert Kilpatrick Clinical Sciences Building, University of Leicester, Leicester Royal Infirmary, Leicester LE2 7LX, UK

## Abstract

Changes in circulating levels of maternal serum transforming growth factor beta-1 (TGF-**β**1), collected from 98 women (AGA) at different gestational ages (10–38 weeks) were measured and comparisons were made between levels in pregnant and nonpregnant controls and also between 10 women with small-for-gestational age (SGA) and 7 with appropriate-for-gestational age (AGA) fetuses. Maternal serum TGF-**β**1 levels at all stages of pregnancy were higher than those in normal healthy nonpregnant adults. The mean TGF-**β**1 levels in SGA pregnancies at 34-week gestation (32.5 + 3.2 ng/mL) were significantly less than those in AGA pregnancies (39.2 + 9.8 ng/mL) while at 38-week gestation, the levels were similar in the two groups (36.04 + 4.3 versus 36.7 + 7.0 ng/mL). This differential change in TGF-**β**1 levels is probably an important modulating factor in the aetiopathogenesis of abnormal intrauterine fetal growth.

## 1. Introduction

Various growth factors such as epidermal growth factor (EGF), insulin-like growth factors I & II (IGF I and II) fibroblast growth factor, and colony stimulating factors are known to be involved in tissue growth and differentiation [1–3]. Alterations in the distribution of EGF receptors have been reported in the placental membranes from intrauterine growth restricted pregnancies [4, 5]. It has been postulated that alterations in various growth factor receptors and their activities may play an important part in the pathogenesis of intrauterine fetal growth restriction (FGR) [6].

Transforming growth factors have been demonstrated at the human fetomaternal interface where it has been suggested that they play a role in the proliferation and differentiation of trophoblasts [7]. Although there are different subsets of this growth factor, transforming growth factor alpha is a homologue of epidermal growth factor and acts through the EGF receptor [6]. Transforming growth factor beta (TGF-*β*), a cytokine, exhibits autocrine, paracrine, and endocrine effects and has been shown to have inhibitory effects on fetal rat hepatocyte and epithelial cell proliferation [8]. In addition studies on TGF expression in placentae from FGR affected and gestational age matched controls concluded that TGF-*β*1 is an important regulator of fetoplacental angiogenesis [9]. A small study found maternal first trimester TGF-*β*1 levels to be higher in pregnancies affected by FGR [10]. Furthermore, levels measured in serum from cord blood of FGR fetuses have been reported to be higher than those of AGA fetuses [11]. Fetal cord blood TGF-*β*1 levels did not, however, correlate with the occurrence or severity of preeclampsia [12]. Other studies failed to find any difference in maternal serum TGF-*β*1 level in normotensive and preeclamptic pregnancies [13] and in placenta from pregnancies complicated by FGR and preeclampsia [14].

While the role of TGF-*β* which modulates trophoblastic proliferation and differentiation in human fetal growth and preeclampsia, as shown in these studies, has not been extensively studied in humans, and indeed the results are conflicting on its precise role in pregnancy, some of the studies were limited either by the small sample size or lacked methodological clarity (e.g., failure to clearly define how the study population was identified). Furthermore, the effect of gestational age on serum TGF-*β*1 levels has not been thoroughly examined.

The objectives of this pilot study were therefore firstly to examine the changes in the levels of transforming growth factor beta-1 (TGF-*β*1) in maternal serum during gestation and secondly to compare the changes in the third trimester in women with small-for-gestational age and appropriate-for-gestational age fetuses.

## 2. Methods

For the first objective (determining changes in TGF-*β*1 levels during pregnancy), a cohort of volunteers was recruited from those attending for ultrasound scan for various reasons. This was a cross-sectional study and each volunteer was sampled once. These were low risk women, nonsmokers, and with no risk factors for preeclampsia, fetal growth restriction, or medical complications. The timing of the recruitment and blood sampling was as follows: 10 (booking ultrasound scan), 20 (routine anomaly scan), 26, 32, 34, 36, and 38 weeks (growth scans for various reasons none of which was for suspected growth abnormality). To be included in this group, the patients had to have delivered an appropriate-for-gestational age baby after 38 completed weeks following a spontaneous or induced labour for postdates. 

To compare the levels of TGF-*β*1 in small-for-gestational age and appropriate-for-gestational pregnancies in the third trimester, 17 volunteers (nonsmokers and matched for age and parity) were recruited from the fetal growth clinic where a majority of the patients were seen on account of a clinical and radiological suspicion of small-for-gestational age at 34- and again at 38-week gestation. They all had babies whose AC < 10th centile on ultrasound scan and no other abnormality and subsequently delivered after 37-week gestation. Pregnant women with complications such as preeclampsia, diabetes mellitus, hypertension, and renal diseases or whose fetuses had evidence of intrauterine compromise such as abnormal umbilical artery Doppler's or severe oligohydramnios (defined as amniotic fluid volume < 3rd centile for gestation) liquor volume were excluded. This pilot population was highly phenotyped as we wanted to concentrate on SGA rather than pathologically small fetuses the subject of follow-up studies. 

Blood samples were also collected from 7 nonpregnant women of similar age in the luteal phase of their menstrual cycle and undergoing sterilisation. They acted as controls for the pregnant group. Signed informed consent was obtained from each of the women from whom samples were obtained. The study was approved by the Leicestershire Ethics Committee. Whole blood samples were collected in serum separator tubes and allowed clotting at room temperature for two hours (to allow complete release of TGF-*β*1) following which they were centrifuged at 1500 ×g for 15 minutes. The separated-serum was stored at −80°C until assays were performed. Assays were only performed on the cohort who met the inclusion criteria. 

Determination of “active” transforming growth factor beta-l (TGF-*β*1) concentration was made by the quantitative sandwich enzyme immunoassay technique (R & D). The procedure of immunoassay was as follows. To 0.5 mL of serum 0.5 mL of 0.25 N acetic acid/10 M urea was added. This was vortexed and then incubated for 10 minutes at room temperature after which the acidified mixture was neutralised by adding 0.5 mL N NaOH/1 M HEPES free acid and vortexing again. The pH of the mixture was then checked to ensure that it was within 7.2 and 7.6. If the pH was outside this range, the volume and corresponding dilution factor of the neutralising reagent was readjusted as required. 

To each well 200 uL of standard or sample was added and then covered with an adhesive strip and incubated for 3 hours at room temperature. Each well was then aspirated and washed four times with a wash buffer. Following this 200 uL of TGF-*β*1 conjugate was added and incubated for 1.5 hours at room temperature. The aspiration and wash step was then repeated and to each well 200 uL of the substrate solution was now added and incubated for 20 minutes at room temperature. 50 uL of the stop solution was then added onto each well and the optical density of each well was determined within 30 minutes using a spectrometer set to 450 nm wavelength. Each sample was measured twice to ensure reproducibility. The interassay coefficient of variability was 5−8%. The results are presented as mean ± standard deviations for the whole group. For samples obtained from the 17 women, unpaired *t*-test was used for comparison between values for the SGA and AGA pregnancies.

At delivery, the babies were classified into either appropriate-for-gestational age (AGA) if their birth weights were above the 10th centile for sex and gestational age or small-for-gestational age (SGA) if their birth weights were below the 10th centile for gestational age and sex (using the nomograms of Wilcox et al.) [15]. 

### 2.1. Statistical Analysis

Statistical analysis of the data was performed using GraphPad Prism version 5.00 for Windows (GraphPad Software, San Diego, CA, http://www.graphpad.com/). As the data were normally distributed, they are presented as mean ± SD throughout.

## 3. Results

A total of 98 pregnant women (i.e., 98 blood samples were collected) were recruited in the cross sectional study: 17 pregnant women for the comparative study (17 samples at 34- and again at 38-week gestation) and 7 nonpregnant controls; in total we analysed 135 serum samples. The mean ages of the cohort were 25 ± 3.6 years. 

In the pregnant women, samples were collected from 10 at the time of their booking ultrasound scan, 11 at 20-week gestation when fetal anomaly scans were performed and 14 at 26-week gestation. The gestational ages in weeks at which the samples were collected in the third trimester were 32 (*n* = 11), 34 (*n* = 21), 36 (*n* = 13), and 38 (*n* = 18). 

The mean gestational age and birth weight in the 98 pregnant women at delivery were 38.7 ± 2.4 weeks and 2945.8 ± 473 grams, respectively. The mean TGF-*β*1 levels during pregnancy (Table 1) fell from 52.7 ± 5.5 ng/mL at 10-week to 46.8 ± 5.5 ng/mL at 20-week gestation and to 40.5 ± 3.8 ng/mL at 26-week gestation. From 32 to 38 weeks, although the levels continued to fall, the differences between values were not statistically significant. The levels were 35.6 ± 5.9 ng/mL at 32 weeks, 33.5 ± 4.6 ng/mL at 34 weeks, 33.2 ± 7.5 ng/mL at 36 weeks, and 33.2 ± 4.7 ng/mL at 38 weeks. This fall in TGF-*β*1 levels during pregnancy was statistically significant (*r* = −0.79, *P* < 0.001).

The mean TGF-*β*1 level in the nonpregnant women was 5.6 ± 1.8 ng/mL (range 3.9–9.3) and was significantly (*P* < 0.02) lower than that in the pregnant women irrespective of gestation. 

**Figure 1:**
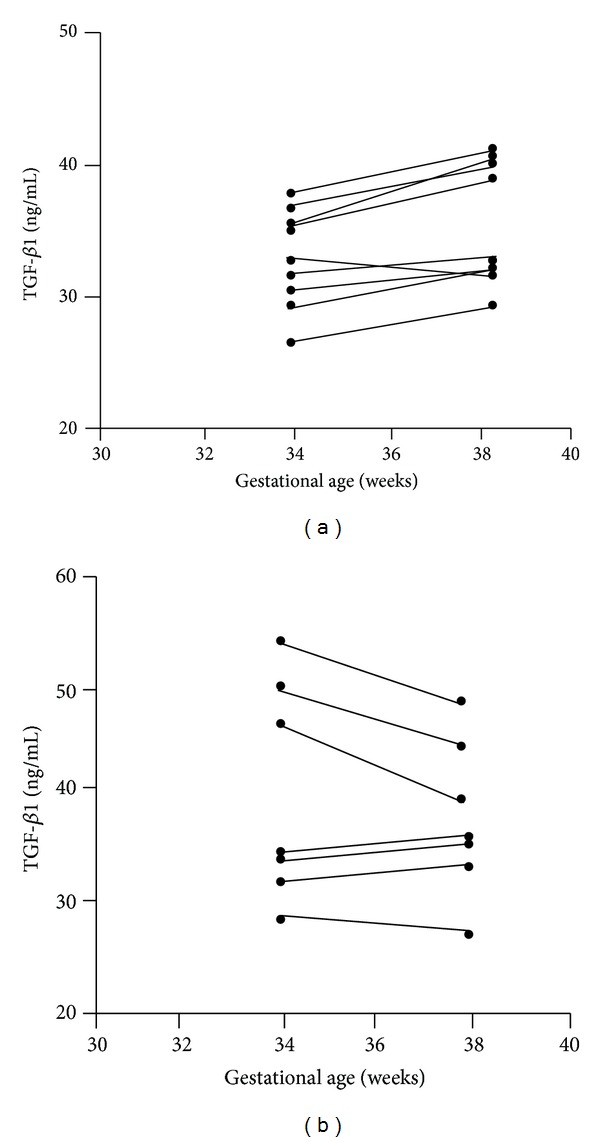
(a) Changes in transforming growth factor beta-1 (TGF-*β*1) levels in SGA pregnancies at 34 and 38 weeks. (*n* = 10; note that the values in two women were very similar). (b) Changes in transforming growth factor beta-1 (TGF-*β*1) levels in AGA pregnancies at 34 and 38 weeks. (*n* = 7).

In the 17 women in whom sampling was performed at 34 and 38 weeks, 10 had SGA babies while 7 had AGA babies.  Table 2 shows the characteristics and TGF-*β*1 levels in these two groups. The mean birth weight in the SGA group was 2159 ± 506 g compared to 3156 ± 301 g in the AGA group (*P* < 0.05). The mean TGF-*β*1 levels at 34 weeks in the women carrying SGA fetuses were 32.5 ± 3.2 ng/mL compared to 39.2 ± 9.8 ng/mL in the women carrying AGA fetuses (*P* < 0.05). At 38 weeks, however, the levels were 36.04 ± 4.3 ng/mL in the SGA group and 36.7 ± 7.0 in the AGA group (NS). Figures 1(a) and 1(b) show the changes in the TGF-*β*1 levels in the two groups between 34- and 38-week gestation. TGF-*β*1 levels increased by 10.9% in the SGA group while in the AGA group there was a fall in TGF-*β*1 level by 6.9%. 

## 4. Discussion

In this study, the values of TGF-*β*1 we obtained in the plasma of pregnant women were much higher than those in nonpregnant controls and those reported in adults (2–18 ng/mL) by Grainger and Metcalfe [16] suggesting that TGF-*β*1 levels rise during pregnancy. These findings were similar to those reported by Hernandez-Valencia et al. [10]. Although the assay technique we used was different from that of Grainger and Metcalfe [16], our range of values from the nonpregnant controls was similar to theirs in adults. It was interesting to note that the pregnancy values were similar to those detected in adults with atherosclerotic diseases [16]. Since there is abundance of TGF-*β*1 at the fetomaternal interface [7] we suggest that the increase in this growth factor during gestation could partly be from the fetoplacental interface.

Hernandez-Valencia et al. [10] also reported a drop in TGF-*β* levels from second to third trimester in AGA pregnancies although this was not significant. In their FGR group, however, there was no change between second and third trimester levels, contrary to our findings of a rise. The main difference between the two studies was that our levels were all measured in the third trimester (i.e., 34 and 38 weeks), whereas Hernandez-Valencia et al. [10] performed their measurements at 28- and 40-week gestation. Furthermore it was not exactly clear how the FGR group was identified in this study.

Transforming growth factor beta stimulates anchorage-independent growth of fibroblast [17], but in most cell types including especially epithelial, endothelial, and haemopoietic cells, it inhibits proliferation. The placenta has a high affinity for TGF-*β* receptors [18] and therefore could be a target tissue for TGF-*β*1. The role of transforming growth factor in the first trimester is thought to be that of regulation of trophoblastic differentiation, proliferation, and invasion [19]. The exact role of this growth factor in the third trimester trophoblast is uncertain but it induces multinucleated cells formation in term trophoblast [7]. Whether TGF-*β*1 has a role in the growth of the fetus is unclear. However, rat fetal hepatocyte cell growth has been shown to be inhibited by TGF-*β* in vitro [6]. Gruppuso et al. suggested that alteration of the effects of TGF-*β* on hepatocyte proliferation in utero may play an important part in fetal growth [6]. 

While TGF-*β*1 levels fell significantly from 10-week through to 26-week gestation, the change in the third trimester in the group as a whole was insignificant. The increase in SGA pregnancies and the paradoxical fall in AGA pregnancies were a very interesting observation. We postulate from these findings that TGF-*β*1 may play a part in the modulation of fetal growth. There are two possible mechanisms by which this modulation may be exerted. The first is via its effects on the proliferation of fetal hepatocytes—the growth of the fetal liver in the third trimester being one of the main determinants of size at birth. The second is via the effects of TGF-*β*1 on placental bed blood vessels. While this may well be a possible mechanism, studies on placental bed biopsies suggest that TGF-*β*1 may not have a role in the pathogenesis of FGR or preeclampsia via these vessels [14] in contrast to studies on placentae [9] which suggest that TGF-*β*1 is a key molecule involved in FGR. The fact that levels were significantly higher in amniotic fluid of preeclamptics [20] would provide further support to a possible role for TGF-*β* in the modulation of fetal growth.

In adults with atherosclerosis, TGF-*β*1 levels have been found to be higher than in adults without vascular disease. Higher levels of this growth factor in SGA pregnancies may therefore be associated with a reduction in perfusion of the placenta and also with a greater inhibition of proliferation and penetration induced directly by higher TGF-*β*1 levels. The consequence of these combined effects is a smaller fetus. We believe that the change in level of this growth factor is more important than the absolute level. We speculate that a steady fall is necessary for satisfactory fetal growth to occur. It would be interesting to perform a study on longitudinal measurements of this growth factor in normal and pathological pregnancies as results from such an observation may provide more information on its potential modulatory role in fetal growth. We would like to emphasise that as the numbers from this pilot study are small, deductions from the results must be made with extreme caution. Furthermore, as the SGA group had no maternal pathology and were probably constitutionally small especially as most were delivered at term, caution must be executed in interpreting our data. Further studies are needed to confirm our findings and whether the observed differences are more marked in severely growth restricted pregnancies. It would also be interesting to measure cord blood TGF-*β*1 levels and to relate these to maternal levels as this may shed more light not only on the maternal fetal gradient but also on a possible origin of TGF-*β*1 if it is not predominantly from the placenta. 

## Figures and Tables

**Table 1:** Changes in transforming growth factor beta-1 (TGF-*β*1) levels during pregnancy when compared to nonpregnant controls.

Gestational age (weeks)	Number (*n* = 98)	TGF-*β*1 levels, ng/mL (mean ± SD)
Nonpregnant (controls), *n* = 7	7	5.6 ± 1.8
10	10	52.7 ± 5.5
20	11	46.8 ± 5.5
26	14	40.5 ± 5.8
32	11	55.6 ± 5.9
34	21	33.5 ± 4.6
36	13	33.2 ± 7.5
38	18	33.2 ± 4.2

**Table 2:** Characteristics of women with SGA and AGA pregnancies and mean TGF-*β*1 levels at 34- and 38-week gestation.

	Small-for-gestational age group (*n* = 10)	Appropriate-for-gestational age group (*n* = 7)
Age at delivery (years)	27.3 ± 5.2	26.4 ± 6.5
Gestational age at delivery (weeks)	38.4 ± 1.0	39.7 ± 0.7
Birth weight (grams)	2159 ± 506	3156 ± 301*
TGF-*β*1 levels at 34 weeks (ng/mL)	32.5 ± 3.2	39.2 ± 9.8*
TGF-*β*1 levels at 38 weeks (ng/mL)	36.04 ± 4.3	36.7 ± 7.0

*P < 0.05 (for SGA and AGA).

Data are shown as mean ± SD.
